# Supplementary material for: Postharvest Drying Techniques Regulate Secondary Metabolites and Anti-Neuroinflammatory Activities of Ganoderma lucidum
Source: Molecules. 2021 Jul 25;26(15):4484. doi: 10.3390/molecules26154484 (PMC8347575; doi:10.3390/molecules26154484)
Supplement: Supplementary file 1 [file molecules-26-04484-s001.zip › molecules-1294706-supplementary.pdf]

Supplementary

Table S1. Metabolites in ethanol extracts from the GL produced by freeze-drying and heat-drying were identified using GC-TOF-MS analysis.

NO.	Compound name	RT (sec)	Freeze-drying (FD)			Heat-drying (HD)		
			FD1	FD2	FD3	HD1	HD2	HD3
**1**	3-Chlorohexane	188.108	6106123	3779119	4026748	4099644	3994594	3642326
**2**	Cyclohexane, 2-propenyl-	200.658	503997	295334	295698	313585	320670	350818
**3**	Trifluoroacetamide, 2TMS derivative	204.600	3380319	2020294	1969645	2122445	2294421	2543599
**4**	Trisiloxane, octamethyl-	214.258	6370932	3999040	4167736	4193233	4217902	5016121
**5**	Methylamine, 2TMS derivative	221.392	287927559	176674701	185093751	175396740	185038634	189532680
**6**	Ethylene glycol, 2TMS derivative	224.233	26711288	16028903	16504767	16920572	17437608	18979239
**7**	Disiloxane, hexamethyl-	226.192	1216157	751113	777863	744110	854025	358399
**8**	2-Bromoethanol, TMS derivative	243.067	205720	133798	134463	128364	136753	134194
**9**	Silanamine, N,N'-methanetetraylbis[1,1,1-trimethyl-	267.883	4079061	2386677	3146979	5248894	5385713	4877802
**10**	Triisopropylsilane	273.183	107967	67134	60169	69163	62142	63133
**11**	O-Ethylhydroxylamine, 2TMS derivative	278.125	382034	229392	239234	250766	244680	214116
**12**	2,2,7,7-Tetramethyl-4-[(trimethylsilyl)methyl]-3,6-dioxa-2,7-disilaoctane	283.708	560752	394420	430608	236122	275645	315396
**13**	2-Methyl-1,3-propanediol, 2TMS	294.433	120087	57471	80467	71621	87873	67422
**14**	3-Hydroxypyridine, 1TMS	322.375	967412	703673	902878	384380	398024	653701
**15**	2-Methyl-1,3-propanediol, 2TMS	323.717	ND	ND	ND	263655	278343	220236
**16**	Decamethyltetrasiloxane	332.983	192820	139765	144698	141481	163488	174141

**17**	Dodecane	334.217	334700	230342	232599	186721	213822	232744
**18**	L-(+)-Lactic acid	336.475	798386	375477	695477	1394181	1282459	1383454
**19**	Acetic acid, [(trimethylsilyl)oxy]-, trimethylsilyl ester	346.200	1125689	1135210	1360959	993802	1042585	867208
**20**	L-Valine, TMS derivative	355.775	310726	220268	345563	2357250	1696683	2588930
**21**	N-Butylamine, DiTMS	361.750	36110	35730	36490	680046	870994	844348
**22**	L-Alanine	365.325	2307030	1558150	2790486	9036011	8506363	9811471
**23**	Hexamethyldisiloxane	366.867	219525	143827	224462	222068	136030	189752
**24**	Hydroxylamine, 3TMS derivative	376.425	11850011	6591775	7119262	7180565	7904158	8855768
**25**	Bis(trimethylsilyl) oxalate	383.525	298490	228772	302723	651397	547188	479599
**26**	3-Furoic acid, TMS derivative	388.033	252316	273202	280968	103299	101023	95184
**27**	Oxalic acid	391.425	1461180	813845	969216	989041	1206821	1060026
**28**	Ethanamine, 2TMS derivative	401.883	371881	373326	418691	3556442	3923372	3748134
**29**	Propanoic acid, 2-methyl-3-[(trimethylsilyl)oxy]-, trimethylsilyl ester	402.783	ND	ND	ND	162307	165981	157440
**30**	Trisiloxane, 1,1,1,5,5,5-hexamethyl-3,3-bis[(trimethylsilyl)oxy]-	410.933	886736	818379	942968	522309	499870	662189
**31**	L-Proline	415.217	ND	ND	ND	1073775	904660	2061214
**32**	Ethylbis(trimethylsilyl)amine	420.217	66599	83076	77263	666819	883028	787652
**33**	3-Hydroxyisovaleric acid, 2TMS derivative	434.267	ND	ND	ND	207477	223921	191848
**34**	D-Valine	439.383	578122	320870	527240	12864758	12854405	13248695
**35**	Trisiloxane, 1,1,1,5,5,5-hexamethyl-3,3-bis[(trimethylsilyl)oxy]-	445.542	36618	28582	28041	29805	38838	37739
**36**	Butanoic acid, 4-	448.733	89075	54806	61895	52449	55180	70071

	[(trimethylsilyl)oxy]-, trimethylsilyl ester							
**37**	4-Aminobutanoic acid, 3TMS derivative	450.417	393693	247663	254544	259445	273856	309008
**38**	Diethylene glycol, 2TMS derivative	454.675	463958	400374	336790	138649	76606	200691
**39**	Trimethylsilyl trimethylsilyl[(trimethylsilyl)oxy]carbamate	455.242	417778	330737	361100	478624	253231	384731
**40**	L-Serine	464.550	210289	182406	321384	923607	931952	1570730
**41**	Ethanolamine, 3TMS derivative	470.525	280157	188376	376457	787649	917915	763291
**42**	L-Norleucine, N-(trimethylsilyl)-, trimethylsilyl ester	472.700	216990	98391	188221	6888075	6228745	5174909
**43**	Glycerol, 3TMS derivative	472.950	396339	328329	554101	2834418	2794669	2724534
**44**	Silanol, trimethyl-, phosphate (3:1)	475.608	8330910	7642310	12129903	30100031	28232189	24945931
**45**	L-Leucine	486.208	274897	145355	257734	6775123	6103178	5393485
**46**	L-Proline, 1-(trimethylsilyl)-, trimethylsilyl ester	490.150	64357	34390	100494	4911196	4490794	3149354
**47**	Glycine	494.917	1306964	866635	1769456	6489618	6174197	6072941
**48**	Succinic acid	495.075	6626719	5533972	8181183	ND	ND	ND
**49**	Silanamine, 1,1,1-trimethyl-N-(trimethylsilyl)-N-[2-[(trimethylsilyl)oxy]ethyl]-	500.658	66277	43441	67533	677825	794162	575741
**50**	Glyceric acid, 3TMS derivative	506.725	78597	81801	99885	76624	82857	66582
**51**	Pyrimidine 2 4-bis (trimethylsilyl)oxy -	512.938	ND	ND	ND	690602	727738	594474
**52**	Butanedioic acid, methylene-,	518.358	159108	119247	140503	150501	138710	136410

	bis(trimethylsilyl) ester							
**53**	L-Serine	523.325	360119	211909	468113	3760576	3756247	2641038
**54**	L-Threonine	538.683	193646	110903	186016	1937383	1871415	1543318
**55**	Glycine, N,N-bis(trimethylsilyl)-, trimethylsilyl ester	544.508	2409143	1367364	1483416	1622552	1679379	1674436
**56**	L-Aspartic acid	557.483	70995	58537	70214	152101	92827	290113
**57**	Decanoic acid, trimethylsilyl ester	572.038	120095	129907	177912	ND	ND	ND
**58**	Malic acid, tris(trimethylsilyl) ester	590.617	3083782	3261472	3759915	4321056	4217631	4343423
**59**	Nonadecane	592.550	160150	116659	123783	82074	104913	127752
**60**	L-(-)-Arabitol	597.350	31443	27467	30498	50246	52372	42853
**61**	3,8-Dioxa-2,9-disiladecane, 2,2,9,9-tetramethyl-	601.583	94775	49111	61287	126946	128026	123108
**62**	L-Aspartic acid, 3TMS derivative	607.075	723855	496363	647209	2066990	2059797	2054309
**63**	L-Glutamic acid	612.283	15706533	14777306	22395131	11699304	12263252	9388913
**64**	Butanoic acid, 4-[bis(trimethylsilyl)amino]-, trimethylsilyl ester	613.090	534650	702307	869963	9384882	8826742	8749041
**65**	Trimethylsilyl 2,3,4-tris[(trimethylsilyl)oxy]butanoate	620.330	15940	14926	16953	53317	54404	50199
**66**	Pipecolic acid, 2TMS derivative	624.125	1234773	1292245	1888010	908098	1045920	626378
**67**	L-Proline	640.117	ND	ND	ND	71917	67496	77029
**68**	1-Oxo-1-(trimethylsilyl)-2-propanone	643.760	115153	123455	143897	35826	38868	32785
**69**	3,9-Dioxa-2,10-disilaundecane, 2,2,10,10-tetramethyl-	646.175	894724	781463	1022579	887187	879259	677067
**70**	L-Glutamic acid, 3TMS derivative	653.967	293254	269610	507742	854728	938052	774390
**71**	Phenylalanine, 2TMS derivative	662.317	156707	104625	171877	1357200	1233538	1095703

**72**	Ethanimidic acid, N-(trimethylsilyl)-, trimethylsilyl ester	670.180	35291	26490	44093	575362	717737	313219
**73**	L-Asparagine	679.717	83626	76672	176963	776561	753189	653060
**74**	Butanoic acid, 4-[bis(trimethylsilyl)amino]-, trimethylsilyl ester	695.400	133829	102792	246207	1626982	1371397	1412727
**75**	L-Rhamnose	709.058	138278	157322	196984	3043299	3086214	3429851
**76**	2-Desoxy-pentos-3-ulose, bis(methoxime),O,O'-bis(trimethylsilyl)-	719.192	196070	162666	231083	692354	651946	696915
**77**	Phosphoric acid, bis(trimethylsilyl) 2,3-bis[(trimethylsilyl)oxy]propyl ester	720.792	537602	329684	435813	507737	682404	724227
**78**	L-Glutamine, tris(trimethylsilyl) derivative	724.400	792603	618157	1567536	2670285	2738244	1873986
**79**	Propanoic acid, 2,3-bis[(trimethylsilyl)oxy]-, trimethylsilyl ester	731.117	ND	ND	ND	21849	18204	18971
**80**	Myo-Inositol	737.233	1817287	2038967	2579830	2497647	3211411	1270948
**81**	9H-Purine, 9-(trimethylsilyl)-6-[(trimethylsilyl)oxy]-	742.333	ND	ND	ND	96021	89505	64524
**82**	Citric acid-tetraTMS	745.183	6811126	6632788	8962139	3945324	3917449	3519911
**83**	Erythrose, MEOX-3TMS	751.675	6733409	5804564	9947044	2134134	2846685	2031262
**84**	1,5-Anhydrohexitol, 4TMS derivative	760.700	928217	812961	1043472	763195	873058	710604
**85**	Myo-Inositol	767.167	653367	594083	742980	613274	857858	381717
**86**	D-(-)-Fructose	770.233	703223	771468	885565	1178051	1255440	1140427
**87**	Gluconolactone (3R,4S,5R,6R)-, 4TMS derivative	774.783	279632	182836	203804	452983	326061	330324

**88**	Silane, tetramethyl-	779.150	48521	55231	57785	ND	ND	ND
**89**	D-(+)-Mannose	781.567	1224106	1103499	1690244	8845106	8571149	9781764
**90**	L-Lysine, 4TMS derivative	785.425	178267	150636	231838	1721490	1733239	1438591
**91**	D-Mannitol	794.742	364999	418850	723517	18603914	16410138	14184646
**92**	Heptasiloxane, hexadecamethyl-	817.300	58815	36120	33408	91968	50661	43791
**93**	D-Gluconic acid, 6TMS derivative	824.033	39680	51379	72959	1158182	927599	1095300
**94**	Palmitic Acid, TMS derivative	828.258	2040669	1255283	1636305	1887926	1621963	1802174
**95**	Inositol	857.492	1999475	1822560	2811621	3461380	3520531	3103439
**96**	Malic acid, 3TMS derivative	869.058	452862	585705	704306	108093	118552	75948
**97**	D-Sorbitol	887.150	ND	ND	ND	41194	49400	41309
**98**	Stearic acid	899.067	1053528	660363	934836	737587	743041	940672
**99**	D-(-)-Ribofuranose, tetrakis(trimethylsilyl) ether	938.000	ND	ND	ND	150050	129448	124617
**100**	Uridine, 3TMS derivative	979.092	687987	288118	295295	896124	1057856	943883
**101**	Gluconolactone	1003.500	438260	283629	299786	1054430	345830	299709
**102**	Inosine, 4TMS derivative	1014.340	149674	89185	108685	508799	532008	428299
**103**	(2,3-Diphenylcyclopropyl)methyl phenyl sulfoxide	1022.540	640879	363946	416217	481125	451800	415618
**104**	Uridine, 3TMS derivative	1033.410	625786	429086	493694	1226341	568594	483369
**105**	Sucrose	1038.580	186334	203265	528541	157634	133772	121714
**106**	D-(+)-Cellobiose	1058.090	120855	115994	138980	149479	148922	111178
**107**	Maltose	1065.120	26578	48324	42769	59828	29004	29456
**108**	D-(+)-Trehalose	1070.190	20233091	18882704	20020118	10380730	13056430	10840066
**109**	Guanosine, 5TMS derivative	1072.130	ND	ND	ND	311722	292393	232792
